# The why, what, where, when and how of goal-directed choice: neuronal and computational principles

**DOI:** 10.1098/rstb.2013.0483

**Published:** 2014-11-05

**Authors:** Paul F. M. J. Verschure, Cyriel M. A. Pennartz, Giovanni Pezzulo

**Affiliations:** 1 Laboratory of Synthetic, Perceptive, Emotive and Cognitive Systems (SPECS), Center of Autonomous Systems and Neurorobotics, Universitat Pompeu Fabra (UPF), Barcelona, Spain; 2 Institució Catalana de Recerca i Estudis Avançats (ICREA), Barcelona, Spain; 3 Faculty of Science, University of Amsterdam, Amsterdam, The Netherlands; 4 Institute of Cognitive Sciences and Technologies, National Research Council, Rome, Italy

**Keywords:** goal-directed behaviour, distributed adaptive control, computational modelling, embodied cognition, reward, decision-making

## Abstract

The central problems that goal-directed animals must solve are: ‘*What* do I need and *Why*, *Where* and *When* can this be obtained, and *How* do I get it?' or the H4W problem. Here, we elucidate the principles underlying the neuronal solutions to H4W using a combination of neurobiological and neurorobotic approaches. First, we analyse H4W from a system-level perspective by mapping its objectives onto the *Distributed Adaptive Control* embodied cognitive architecture which sees the generation of adaptive action in the real world as the primary task of the brain rather than optimally solving abstract problems. We next map this functional decomposition to the architecture of the rodent brain to test its consistency. Following this approach, we propose that the mammalian brain solves the H4W problem on the basis of multiple kinds of outcome predictions, integrating central representations of needs and drives (e.g. hypothalamus), valence (e.g. amygdala), world, self and task state spaces (e.g. neocortex, hippocampus and prefrontal cortex, respectively) combined with multi-modal selection (e.g. basal ganglia). In our analysis, goal-directed behaviour results from a well-structured architecture in which goals are bootstrapped on the basis of predefined needs, valence and multiple learning, memory and planning mechanisms rather than being generated by a singular computation.

## 1. Introduction

Instrumental actions can be either habitual or goal-directed. In order to label an action goal-directed, it must satisfy two requirements. First, the agent must display knowledge of the *causal efficacy* of its own actions and their *outcomes* given the current *state* or *context*. Second, the agent must select and regulate its behaviour using *goal representations*, e.g. internally generated representations of desired action outcomes. In other words, such deliberate action is directed towards specific states of the external world, i.e. goals, motivated by states of the internal environment and mediated by internal representations of these goals. Goal-oriented action expresses agency, mental states and is intentional following the nineteenth century philosopher Franz Brentano. A fundamental nature challenge in psychology and neuroscience is to develop a coherent explanation of goal-directed action. This has turned out to be difficult as the history of the study of behaviour illustrates. During behaviourism, constructs such as ‘intention’ or ‘goal’ with their associated teleology were seen as unscientific and were thus eliminated. With the cognitive revolution, these notions were again incorporated in the terminology of mind and brain [1] but the mechanistic functioning and neuronal underpinnings of goal-directed behaviour remained largely unknown. In the perspective of symbolic artificial intelligence (AI), knowledge and goals are organized following a principle of rationality: ‘… if the system wants to attain goal *G* and knows that to do act *A* will lead to attaining *G*, then it will do *A*. This law is a simple form of rationality that an agent will operate in its own best interest according to what it knows’ [2, p. 49]. In this case, however, the problem was that ‘goals’, ‘knowledge’ and ‘actions’ were all defined *a priori* and the explanation of cognition limited itself to the operations that could be performed on these symbolic representations. No commitments were made to their etiology and the question thus becomes how an agent can autonomously acquire or define these core elements of cognition [3].

The explanation of goal-oriented behaviour is not only of interest because of the complexity of the associated phenomena but also because it is at the edge of phenomenology and science. As much as there is a ‘hard problem’ in the explanation of consciousness [4,5], we can argue that a similar explanatory gap exists in understanding goal-oriented behaviour, it also critically depends on inferences by the observer on the beliefs an organism entertains with respect to its goals and tasks. The fundamental challenge is to explain this phenomenon while acknowledging the full richness of its intentional aspects, avoiding the behaviourist fallacy. Conversely, our explanation should include the genesis of goals as opposed to assuming them *a priori* to avoid the trap of symbol grounding of traditional AI. This paper tries to directly answer these challenges by addressing the notion of goals and their role in cognition and action from an integrative perspective building on three components: (i) an evolutionarily motivated hypothesis on brain function and action; (ii) an embodied theory of mind and brain called distributed adaptive control (DAC), and (iii) a detailed system-level analysis of the neuronal substrates of goal-oriented behaviour in the rodent. In this integration, we take as our specific question whether goals should be seen as single scalar functions against which behaviour is optimized or whether they are multi-dimensional processes that emerge from the interaction across a number of perceptual, affective, cognitive and motor systems (see also [6]). We advocate the latter interpretation and show that goals and their impact on ongoing and future behaviour should be seen as resulting from a process that plays out at multiple levels of the neuraxis following distinct principles.

Goal-directed behaviour is unique and distinct from other forms of control such as innate reflexes and habits in the sense that it does not prescribe a specific operation or procedure but rather the end state that an operation should achieve. Goal-directed choice permits an agent to escape from stereotyped interactions with a predictable environment and flexibly and rapidly adapt to complex and dynamic internal (e.g. motivational) and environmental conditions using abstract allocentric procedures and prospection. This fact is epitomized in experimental procedures such as reward devaluation [7]. Here, a rat is first trained to press a lever to obtain a food reward. After reaching the learning criterion, the rat is given the same reward but now ‘devaluated’, e.g. coupled to a nausea inducing treatment. When the rat is subsequently placed again in front of the lever, it can either display the habit of the previously acquired lever-pressing action or display goal-oriented (in)action by avoiding to press the lever. Rats are able to suppress the acquired response. This example illustrates the two hallmarks of goal-directed choice: agency and intentionality. It also shows that goal-directed choice is a systems property that depends on a number of tightly coupled processes including perception, motivation, emotion, cognition and action. It cannot be localized to a central ‘goal nucleus’ in the brain but rather depends on the interplay of a number of mechanisms realized in several brain areas. As a result, understanding goal-directed choice requires a *systems-level architectural* treatment and recent advances have clarified the possible contributions of specific components of this architecture [8,9]. However, it is less clear how the contributions of these different brain areas are orchestrated to generate actions that lead to goal achievement in the real world and how goal-directed mechanisms coexist with other non-goal-based ones. Here, we propose a systems-level solution to the challenge of goal-directed choice from combined experimental neurophysiological and theoretical perspectives.

In order to create structure in the tangle of neuronal processes and sub-processes that make up the brain and their multi-level organization, we need to define unambiguously what the overall function of this system is. Here, we follow Claude Bernard and Ivan Pavlov in defining the brain as a control system that maintains a metastable balance between the internal world of the body and the external world through action. The question thus becomes: ‘does it take to act?’ We propose that in order to act in the external world, the brain needs to optimize a specific set of objectives which are captured in answering the questions: ‘*Why* do I need to act? *What* do I need? *Where* and *When* can this be obtained and *How* do I get it?’ These questions harbour a complex set of computational challenges that can be defined as the *H4W problem* [10]. In short, an animal needs to determine a behavioural procedure to achieve a goal state (the How of action), which in turn requires defining the ‘Why’ (the motivation for action in terms of needs, drives and goals), ‘What’ (the objects and their affordances in the world that pertain to these goals), ‘Where’ (the location of objects in the world, the spatial configuration of the task domain and the location and confirmation of the self) and ‘When’ (the sequencing and timing of action relative to the dynamics of the world and self). We propose that goal-oriented action in the physical world emerges from the interplay of the different processes subserving H4W. Action in a social world would also require processing ‘Who’, but this aspect will not be dealt with here [11].

Each of the Ws can be seen as a specific objective that the brain must satisfy, to which it designates a large set of sub-objectives of varying complexity, which are laid out in parallel across different levels and scales of organization of the central nervous system. At a first level, the brain must assess the motivational states derived from homeostatic self-essential variables. These motivational states in turn need to be prioritized so that goals can be set: this is the ‘Why’ problem, requiring the modulation of associated behaviour systems. Next, a second layer of control is called for to classify, categorize and valuate states of the world, to identify the spatial layout of the task, including the agent itself, and the dynamics of the task and its affordances: ‘What’, ‘Where’, ‘When’. Lastly, these labelled multi-modal states are grouped in sequences around prioritized goals; for example, in a rodent navigation set-up, to go towards and push a lever, placed at the northeast corner of the environment, given that the cue signal has appeared. At this stage, the ‘How’ has been generated and expressed. Using the accumulated spatio-temporal knowledge of the task and the self in which goal pursuit is framed, a procedural motor strategy (‘How’) can be composed and its elements selected from the set of available options to achieve a goal state. We propose that the H4W framework outlined above is an exclusive set of processes that captures the essential brain mechanisms that mediate and control instrumental interaction with the physical world. We will now show how H4W maps onto a biologically grounded cognitive architecture that will further facilitate to show how we can make sense of the intricate neuronal substrates of goal-oriented behaviour.

### (a) The distributed adaptive control theory of mind and brain

**Figure 1.**
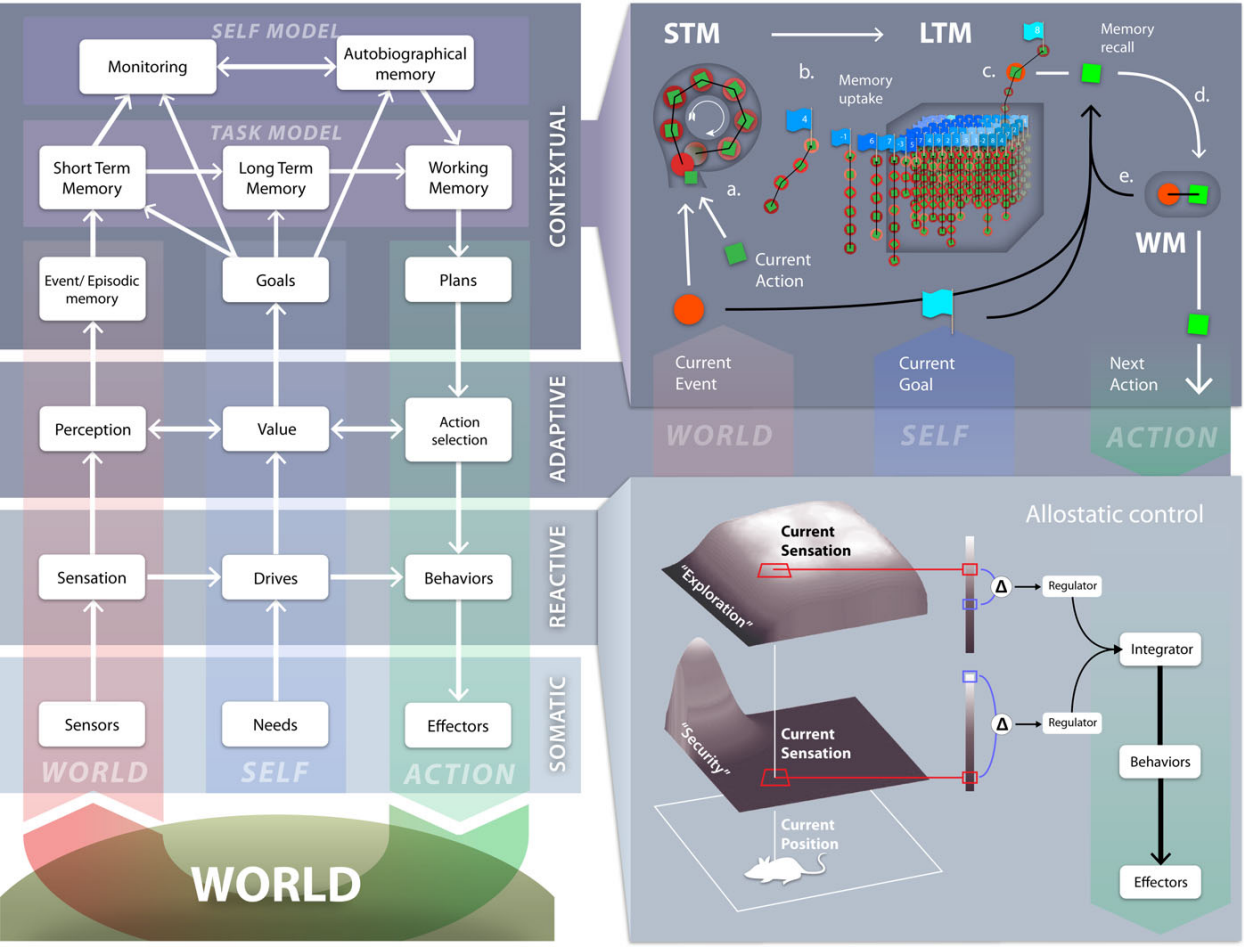
The DAC theory of mind and brain (see [10] for a review). Left: highly abstract representation of the DAC architecture. DAC proposes that the brain is organized as a three-layered control structure with tight coupling within and between these layers distinguishing: the soma (SL) and the reactive (RL), adaptive (AL) and contextual (CL) layers. Across these layers, a columnar organization exists that deals with the processing of states of the World or exteroception (left, red), the self or interoception (middle, blue) and action (right, green). See text for further explanation. The reactive layer: the RL comprises dedicated behaviour systems (BS) that combine predefined sensorimotor mappings with drive reduction mechanisms that are predicated on the needs of the body (SL). Right lower panel: each BS follows homeostatic principles supporting the self-essential functions (SEF) of the body (SL). In order to map needs into behaviours, the strength of the essential variables served by the BSs, SEFs, have a specific distribution in task-space called an ‘affordance gradient’. In this example, we consider the (internally represented) ‘attractive force’ of the home position supporting the security SEF or of open space defining the exploration SEF. The values of the respective SEFs are defined by the difference between the sensed value of the affordance gradient (red) and its desired value given the prevailing needs (blue). The regulator of each BS defines the next action as to perform a gradient ascent on the SEF. An integration and action selection process across the different BSs forces a strict winner-take-all decision that defines the specific behaviour emitted. The allostatic controller of the RL regulates the internal homeostatic dynamic of the BSs to set priorities defined by needs and environmental opportunities through the modulation of the affordance gradients, desired values of SEFs and/or the integration process. The adaptive layer: the AL acquires a state space of the agent–environment interaction and shapes action. The learning dynamic of AL is constrained by the SEFs of the RL that define value. The AL crucially contributes to exosensing by allowing the processing of states of distal sensors, e.g. vision and audition, which are not predefined but rather are tuned in somatic time to properties of the interaction with the environment. Acquired sensor and motor states are in turn associated through the valence states signalled by the RL. The contextual layer: the core processes of the CL are divided between a task-model and a self-model. The CL expands the time horizon in which the agent can operate through the use of sequential short-term and long-term memory (STM and LTM) systems respectively. These memory systems operate on integrated sensorimotor representations that are generated by the AL and acquire, retain and express goal-oriented action regulated by the RL. The CL comprises a number of processes (right upper panel): (a) when the discrepancy between predicted and encountered sensory states falls below a STM acquisition threshold, the perceptual predictions (red circle) and motor activity (green rectangle) generated by AL are stored in STM as a, so-called, *segment*. The STM acquisition threshold is defined by the time-averaged reconstruction error of the perceptual learning system of AL. (b) If a goal state (blue flag) is reached, e.g. reward or punishment, the content of STM is retained in LTM as a sequence conserving its order, goal state and valence marker, e.g. aversive or appetitive, and STM is reset. Every sequence is thus labelled with respect to the specific goal it pertains to and its valence marker. (c) If the outputs generated by the RL and AL to action selection are sub-threshold, the AL perceptual predictions are matched against those stored in LTM. (d) The CL selected action is defined as a weighted sum over the segments of LTM. (e) The contribution of LTM segments to decision-making depends on four factors: perceptual evidence, memory chaining, the distance to the goal state and valence. Working memory (WM) of the CL is defined by the memory dynamics that represents these factors. Active segments that contributed to the selected action are associated with those that were previously active establishing rules for future chaining. The self-model component of the CL monitors task performance and develops (re)descriptions of task dynamics anchored in the self. In this way, the system generates meta-representational knowledge that forms autobiographical memory. This aspect of the DAC CL is not further considered in this paper.

To map the functional H4W hypothesis to potential neuronal substrates of goal-oriented behaviour, we first decompose it in terms of a hypothetical neuronal architecture of the mammalian brain called DAC^1^ (figure 1, see [10] for a review). The DAC theory emphasizes that perception, emotion, cognition and action are realized through an integrated control system that is both embodied and situated in the real world. In this perspective, goal-directed choice is not a monolithic component but depends critically on the interaction between several layers of control, each of which uses specific information (e.g. motivational and sensory versus memory and prospection) to generate and maintain goal representations at varying levels of abstraction that cooperate and compete for the control of action [13]. Thus, from the DAC perspective, drives and goals are necessarily organized in a hierarchical fashion, starting with the concrete needs defined by the physical instantiation of the agent, i.e. the nutrients required to maintain the body, to the abstract goals of reaching specific but also abstract goal states, such as for example, having dinner in a specific restaurant or becoming an expert on Byzantine culture. The latter are seen as being bootstrapped on the basis of the former. Below we introduce the layered organization of DAC and discuss how it constitutes a coherent architectural solution to the H4W problem of goal-directed behaviour.

The s*omatic level* (SL) of DAC designates the body itself and defines three fundamental processes: *exosensing* of states of the environment, *endosensing* of states of the body or essential variables of survival, defining *needs* and *actuation* through control of the skeletal-muscle system*.* Behaviour is defined as a change in the confirmation and/or position of the SL.

The *reactive layer* (RL) of DAC supports the basic functionality of the SL and generates control signals that drive and modulate the engagement of higher control layers and their epistemic functions [14] (figure 1, lower right panel). These sensorimotor loops are organized in fundamental and opposing behaviour systems that support basic needs such as fight, flight [15], seek and play [16]. Every behaviour system is homeostatic but importantly their collective properties are in turn regulated by an integrative *allostatic* loop [17,18]. This allostatic orchestration (see figure 1) is critical to goal-oriented behaviour because both at a behavioural and physiological level, different homeostatic subsystems are in a competitive relationship and priorities and hierarchies must be established on the fly dependent on internal needs, external threats and opportunities. In order to control behaviour, the states of the behaviour systems have a specific neural representation that distributes them across space, called an *affordance gradient*, such as the ‘attractive force’ of the home position or the ‘repellant force’ of a predator, that thus implicitly encodes action in an egocentric frame of reference [19]. DAC thus sees the reactive agent as operating in a composite behavioural field defined by the dominant behavioural subsystems and their internal representation of affordance gradients. This mechanism links well to the idea of force-field-based control as used in the individual behaviour systems (BS) of RL. This idea has its roots in Gestalt psychology [20] and is a standard approach towards robot navigation [21] but generalizes towards optimal path planning in mobile robots [22] and social interaction in humanoid robots [23].

The *adaptive layer* (AL) of DAC extends the predefined need-reducing sensorimotor loops of the RL with value-dependent acquired sensor and action states. It allows the agent to escape from the strictly predefined and instantaneous reflexes of RL through learning [24,25]. The AL constructs a state space encoding of both the external (world) and internal (self) *a priori* unpredictable environment and shapes the amplitude-time course of the predefined RL reflexes. It crucially relies on distal sensors, e.g. vision and audition. The acquired sensor and motor states are in turn associated through the valence states triggered by the RL, following the paradigm of classical conditioning where initially neutral or conditioned stimuli (CS) obtain the ability to trigger actions, or conditioned reponses (CR), by virtue of their contingent presentation with intrinsically motivational stimuli or unconditioned stimuli (US) [12]. In particular, the AL explains the principles underlying the two-phase theory of classical conditioning [26] combining the, so-called, non-specific and specific learning systems into one integrate behavioural architecture. DAC's AL predicts that the former plays a key role in value-dependent stimulus identification in addition to the preparation for action, while the later is dependent on the former and critical for timing rather than ‘consumption’ [27]. The AL allows the agent to overcome the predefined behavioural repertoire of the RL and to engage an *a priori* unpredictable world, solving the notorious symbol grounding that led to the demise of classical AI [3,28].

The learning dynamics of the AL are defined in terms of minimization of the prediction error between acquired and encountered states of the world relying on local Hebbian learning [24]. This approach has been rephrased in a general formal framework called *correlative subspace learning* (CSL) where the associations between perceptual states and action are mediated via value representations and perception-value associations are formed on the basis of both perceptual and behavioural prediction [25]. CSL is consistent with ‘predictive brain’ frameworks and ‘free energy minimization’ principles [29,30]. Importantly, DAC demonstrates these principles within an embodied cognitive architecture. Experiments with AL have three important consequences for our understanding of goal-oriented behaviour. First, prediction-error minimization is a multi-scale process that needs to operate in concert with dedicated processes for behaviour control and exploration provided by the RL. Second, prediction-based learning is crucial in real-world behaving systems in order to counteract *behavioural feedback*, i.e. non-neuronal feedback resulting from both the high spatio-temporal correlation in sensory streams combined with experience-dependent biased sampling of the sensory space [13,24]. Third, the strict distinction between retrospective model-free and prospective model-based decision-making is misleading in the sense that in both cases, the state space on which decision-making operates is defined based on models albeit of varying spatio-temporal complexity [31].

AL extends the need-reducing sensorimotor loops of RL into acquired sense-valence-act triplets that include *a priori* unknown but now acquired states of the world and the self. This adaptation occurs in a restricted temporal window of relatively immediate interaction, i.e. up to about 1 s, which is the range in which most forms of classical conditioning operate. DAC predicts that this temporal boundary is dependent on the characteristic time constants of cerebellar learning loops [32]. However, in order to escape from the ‘now’ and value-dependent interaction with the world and develop goals in an expanded spatio-temporal window, more advanced memory systems must be engaged: the contextual layer (CL).

The CL of DAC acquires, retains and expresses behavioural plans by combining sequences of AL-defined sensorimotor states with goals in a value-dependent way (figure 1, upper right panel). The CL comprises a dual representational system: one for representations of tasks and the other for representing the self. The former comprises systems for short-term, long-term and working memory (STM, LTM and WM, respectively), while the latter combines a monitoring system of the Task Space with an autobiographical memory. The task space memory systems allow for the formation of sequential representations conditional on the goal achievement of the agent. CL behavioural plans can be recalled through sensory matching and internal chaining among the elements of the retained memory sequences (see figure 1). The dynamic states that this process entails define DAC's WM. The CL organizes LTM along behavioural goals and previous studies have assessed that this together with valence labelling of LTM segments is required, in order to obtain a Bayesian optimal solution to foraging problems [3]. Goals are formed through the integration of sensorimotor states with the termination conditions of behavioural patterns defined through need-reduction and value systems of the RL and AL, respectively. Goals are initially defined in terms of the drives that are guiding the behaviour systems of the RL such as finding a food item, or solving an impasse, i.e. flight, and the valence that they are associated with at the AL. Through learning, goal states can expand to include the sensorimotor states that brought about the change in drive and valence forming a multi-modal construct. Goal states, as termination points of acquired behavioural procedures, together with the behavioural sequence itself exert direct control over how decision-making and action selection is performed.

DAC proposes a four-factor decision-making model that uses: *perceptual evidence*, *memory* chaining, the predicted distance to the *goal* state, or goal fidelity, and the expected *value.* This four-factor decision-making model of CL predicts that decision-making will display both goal and behavioural procedure fidelity [33,34]. This means that potential actions are weighted both with respect to their distance to the current goal state as measured in the number of events between the memory element and the occurrence of the goal state, and whether they form part of an active behavioural sequence, where active sequences are triggered through perceptual evidence and memory priming processes. This expansion of the decision-making towards goals frees the agent from acting in the restricted temporal window of the AL, its egocentric frame of reference and its dependence on immediate sensory states. Rather, memory allows for the organization of behaviour along allocentric coordinates, prospection and symbolic representations that do not depend on available sensory states. This transition towards allocentric goal-oriented navigation leads to robust robot multi-modal (proximity sensors, chemosensing and vision) foraging including recovery from kidnapping and noise [35] and optimal maze navigation in the presence of distracters [36]. These robot experiments with DAC show that the ability to navigate using allocentric coordinates is closely coupled with the use of goals and goal fidelity. Moreover, the notion of goal fidelity suggests that goals cannot be seen as single scalar values that act as discrete organizers of action (e.g. in the form of a single reward signal), but are defined through the confluence of congruent sensory, motor and value information at the termination points of behavioural sequences, i.e. the sequences formed in LTM. These goal states also exert an implicit influence on decision-making by defining a goal-dependent metric that measures the relevance of specific memory elements to the current task (i.e. goal fidelity). When action depends solely on perceptual evidence and egocentric representations, any variation of these input states (due to occlusions, noise, movement-induced variation, etc.) translates into variability in action selection leading to a nonlinear amplification of behavioural variability and thus the deviation from previously executed and acquired trajectories. This so-called *behavioural entropy* precludes the agent from reusing acquired egocentrically represented behavioural plans because predicted sensory states will not match encountered ones. To demonstrate how allocentric goal-oriented behaviour enhances fitness by overcoming behavioural entropy, experiments were performed where egocentric action encoding was compared with an allocentrically defined movement vector that points from the current position of the agent to the location of a next landmark, i.e. allocentric goal-based action [37,38]. Using a range of maze learning benchmarks, it was shown how this approach leads to robust maze navigation and learning even when significant noise is applied to the motor output [33]. This study also illustrates the relationship between taxon versus route navigation strategies [39] in a robotic system. The reactive egocentric control system can fulfil the agent's needs when the information from the goal site can be directly detected, there is a direct path between current and goal positions (taxon-based strategy) and approaching it does not conflict with any other behaviour system. However, when a taxon-based strategy fails or relevant landmarks cannot be detected, the contextual control system is required to generate a route using an allocentric navigation method relying on acquired goal states. On the basis of these observations, DAC predicts that AL is focused on real-time egocentric interaction with the world emphasizing interval timing, while CL abstracts its processing towards symbolic events and their order supporting allocentric interaction with a task.

The self-model of the CL includes monitoring and autobiographical memory systems. The former prevents the consolidation of behavioural sequences that are not causally related to goal achievement defining an agency condition for CL memory formation. The later is an episodic memory system that memorizes self-generated goal-oriented behaviours and its context and centres on the self and its agency as opposed to the task and the world in which it is realized.

In summary, the DAC theory introduced proposes that the elements of H4W are processed at each layer of the neuraxis organized along World (What, Where, When), Self (Why) and Action (When, Where, How). Here, ‘Where’ and ‘When’ appear twice to emphasize the distinction between Self and external objects. As we advance along this hierarchy, the agent becomes less dependent on immediate states of the world and relies more on memory increasingly constrained by states of self, from needs to values to goals, in an expanding spatio-temporal horizon. In this hierarchy, the RL need-reduction systems define the value systems of the AL, which in turn constrain the behavioural plans formed by the CL. By combining need reduction and value with the sensorimotor states of the task at hand, goals emerge as CL-based representations of termination points of acquired behavioural procedures that can operate in an allocentric frame of reference freeing the agent from the here-and-now and propelling it towards a goal-oriented future.

## 2. The neurobiology of drives and goals according to the H4W-DAC taxonomy

**Figure 2.**
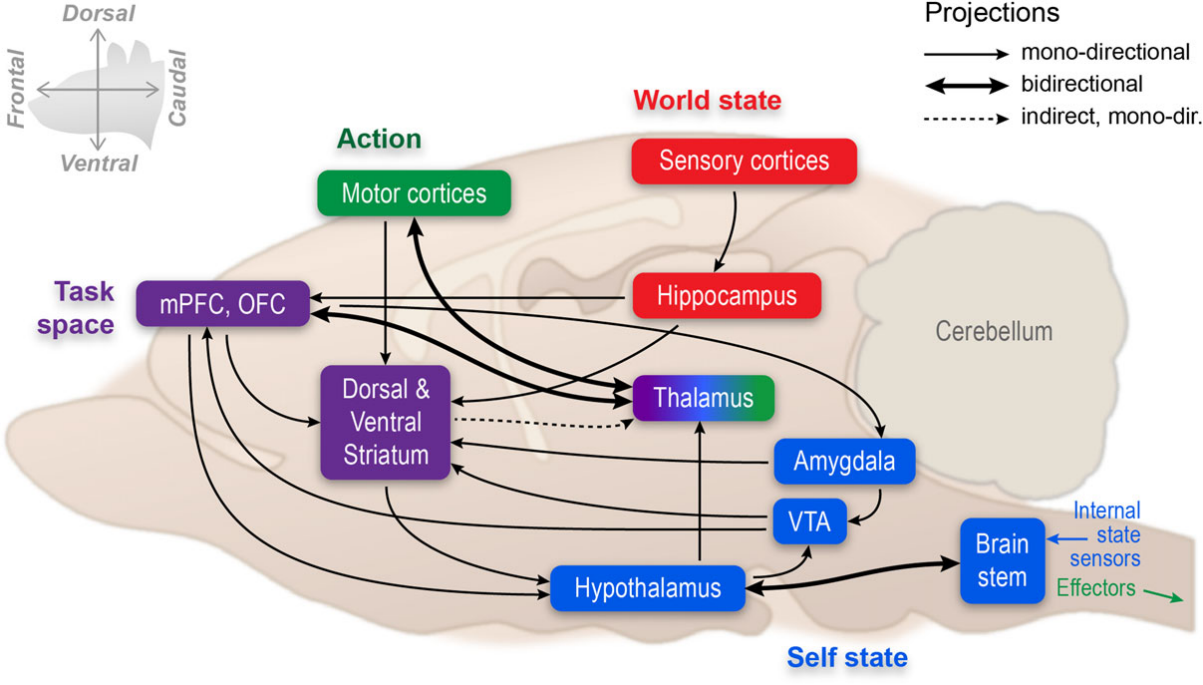
Proposed brain architecture representing the neuronal substrate of goal-directed behaviour and its relation to the neurorobotic DAC architecture. The hippocampal formation is proposed to code the organism's world state space (red), the prefrontal cortex (especially its medial and orbitofrontal aspects) to represent task space (i.e. rules, constraints, goals and values of cues and action options, purple) and the striatum (and downstream structures of the basal ganglia) to mediate action selection. In this scheme, the hypothalamus and brain stem contain sensor systems monitoring homeostatic variables and providing information about the motivational needs of the organism that define the pursuit of needs and goals (blue). The arrow from striatum to thalamus represents an indirect projection. The hypothalamic efferents are modelled after those traced for the lateral hypothalamus in relation to feeding behaviour and do not apply to hypothalamic areas in general. For the sake of clarity, the scheme's anatomic connections are by no means complete. For instance, outputs from prefrontal cortex and basal ganglia to the brain stem, or several afferent inputs to amygdala and VTA, have not been included, while several hypothalamic nuclei project directly to ventromedial prefrontal areas. Sensory inputs reach the hippocampus via intermediate stations (parahippocampal areas; not shown) and are supplemented with frontal cortical inputs converging on these intermediate areas. Furthermore, the motor cortices are meant to include premotor, supplementary motor and frontal oculomotor areas (based on [40–42]).

The H4W taxonomy and its realization in the DAC architecture suggest a three-level organization of the central nervous system. Below we discuss how each of these three levels of organization is neurally implemented in the mammalian brain, reviewing mainly rodent research (figure 2).

### (a) Reactive layer: neural mechanisms encoding drives in relation to survival and reproduction

Perhaps the most well-known brain region for controlling motivational drives is the hypothalamus, a phylogenetically ancient, diencephalic structure well connected to sensor and actuator systems in lower CNS centres such as the brain stem, spinal cord and autonomic ganglia. For goal-directed behaviour, both the sensor and effector functions of the hypothalamus are critical. For instance, the preoptic area contains thermosensitive neurons that, at the same time, are involved in the generation and dissipation of body heat [43]. The supraoptic nucleus harbours cells that gauge the osmolality of blood plasma [44], and at the same time this nucleus maintains homeostasis of blood salt levels. Similarly, hypothalamic cell groups are thought to monitor nutrient levels and guard the body's energy balance, while others regulate sexual, maternal and aggressive behaviour as well as sleep (e.g. [45–49]). This list of hypothalamic sensor–actuator functions is by no means exhaustive and can be supplemented with numerous brain stem-medulla nuclei that are often positioned even closer to internal sensors and effectors (e.g. the monitoring and regulation of food intake, respiratory and cardiovascular reflexes by the nucleus tractus solitarius, vagal nuclei and connected cell groups). In addition, these functions address not only homeostatic regulation, but also allostasis, referring especially to responses to challenges that require system-wide, dynamic adaptation and predictive regulation in anticipation of upcoming homeostatic disturbances [17,18].

For goal-directed behaviour, a first point to note is that drives and their associated behavioural expression, such as aggression, thirst, hunger, sex, sleep and safety, are determined in basic form at the level of the hypothalamus together with lower-order structures [50], analogous to the functioning of the RL in DAC. A drive arises from the discrepancy between a read-out of a homeostatic parameter (e.g. blood sugar level) and an optimal set point, although for some types of drives the neural basis underlying this comparison is not that clear yet (e.g. for ‘sleep pressure’, e.g. [18,51]). Second, a definition of drive purely in terms of internal homeostasis would be too limited. For instance, organisms will explore novel environments and objects even if they thereby leave their shelter and decrease their own safety level, while no explicit reward is ensured. It is still largely unknown how such a drive for novel information or exploration (or reduction of uncertainty [52–55]) would be realized by the brain, although some higher-level control systems have been implicated (e.g. prefrontal cortex and hippocampus; see below). In addition, the question is whether we need to distinguish different levels of drives from ‘basic and innate’ to ‘derived and acquired’ arranged in a hierarchical fashion, with the associated question of how many of such drives must be identified. It is especially with an eye to optimizing the dynamic equilibrium between the multiple competing needs of the organism and the dynamics of its environment that the notion of allostasis has been proposed. Third, our relatively advanced knowledge of homeostatic ‘reflexes’ (i.e. automated sensor–effector reactions to specific disturbances of homeostatic equilibria) has done little to inform us about mechanisms to *prioritize* particular drives over others. For instance, if an animal is hungry, thirsty and exhausted at the same time, it is less than clear how the brain prioritizes a particular drive as being targeted for satisfaction and deals with conflict resolution. A chosen priority will not only depend on the strength of the drive but also on the context of available options, confidence in available solutions, etc. prompting the organism to estimate how likely a goal (e.g. quenching of thirst) can be achieved given the environment and its affordances (dry or rainy). The DAC architecture solves this challenge relying on allostatic control in the service of need reduction (figure 1).

Returning to the notion of *different levels* for organizing goal-directed behaviour, we will review evidence implying higher brain structures in the neural implementation of what we have described as its core characteristic: the prospective evaluation of possible *state* (situation) and *action outcomes* rather than fixed stimulus–response rules. As mentioned in the Introduction, goal-directedness implies that the organism has implicit or explicit knowledge about its actions being causal in the chain of events leading to the desired goal, i.e. agency. Furthermore, if the value/utility of a goal changes, for instance because of a change in motivational state, the organism should refrain from persistently conducting a fixed stimulus–response sequence or habit, but must adjust its response according to the change in value [7]. Given this definition of goal-directedness and the limitations of hypothalamic-brain stem systems in this respect owing to their reliance on fixed action patterns, there is a need for ‘higher’ systems to be informed about drives expressed by these ‘lower’ reactive levels. This upward projection of the drive state implements a reactive-to-adaptive, flexible type of organization that grounds the affective component of goals and can be realized, among others, through hypothalamic projections to the thalamus, prefrontal cortex, hippocampus and related structures [41].

### (b) State representations conducive to goal-directed behaviour: hippocampus

Currently, it is becoming clearer that, when an animal is strongly committed to obtaining a particular goal, information processing in many brain areas is simultaneously affected by inputs that are predictive of, or conducive to, this goal. This effect is more widespread and pervasive than could be captured by the simplification that ‘reward centres’ in the brain are activated. Even structures such as posterior parietal cortex and primary sensory cortex (A1, V1) are deeply affected by associative stimulus–reward and action–reward learning [56–62]. Despite this ubiquity, there are good arguments to highlight the roles of hippocampal-prefrontal systems in forming *state representations* that can be used by action selection systems executing goal-directed behaviour. Put succinctly, when the needs of an agent (‘Why’) have been set at the level of the hypothalamus and brain stem, representations of the state of the world (including the agent's own state) are required to determine where and when this need may be satisfied, and through which particular object (‘What’) within a feasible spatio-temporal range (e.g. an apple to satisfy the need for particular nutrients).

Over the past decades, two classical views of the hippocampal system have been elaborated, both of which are currently in need of updating with respect to goal-directed behaviour. The first view holds that the hippocampus codes an agent's position in space, as inspired by the Cognitive Map theory of Tolman [63] as well as the body of place-cell research initiated by O'Keefe & Dostrovsky [64]. The second notion accounts for hippocampal function as a ‘recorder’ of experience—an organ for the formation of episodic memories that would be transferred, with the passage of time, to neocortical sites where also generalization (semanticization) of memory may take place [65]. The update required on the first view is that a large body of recent research indicates that the hippocampus codes not only for an agent's self-location, but also for specific objects and events—e.g. neutral environmental cues such as odours, and cues that predict reward, as well as the time spent in a situation conducive to goal pursuit [66–70]. Moreover, the representation of the task seems to follow a multiplexing of input streams combining sensory, location and action information at both the input and memory stages of hippocampal processing wood [66,71–73]. This latter observation is in line with a prediction from the DAC theory, viz. that goal-oriented behaviour is constructed from conjunctive sensorimotor couplets (figure 1). If true, this would mean that modulation of sensory cues should lead to a significant change in the population response of hippocampal neurons [74]. This effect was indeed directly observed in, so-called, rate remapping in environments that were morphed [75] and abolished when sensory cues to the hippocampus are removed [76].

The update on the second notion of the recording of experience is that the hippocampus has turned out not only to record (i.e. encode and store) spatial experiences, but also—and more generally—chains of associated events and sequences of motor actions [77–79]. Indeed, the DAC theory predicts that sensorimotor couplets are constructed and combined into sequential representations in STM. The hippocampus seems to display both sensory and motor features. How these chains are formed is unknown, but in the case of motor sequences the information is hypothesized to reach the hippocampus via thalamocortical sensory systems (e.g. somatosensory, proprioceptive and vestibular), where novel information can be associated with retrieved memories to expand these into longer chains. Importantly, the hippocampus appears only to be required for longer, or more complex, sequences as more simple stimulus–response associations (and ensuing habits) can be acquired via the dorsal striatal system [80,81].

In addition to storing information, the hippocampus is able to retrieve previously stored information and to self-generate internal sequences of cell activity that are subsequently used to map novel environments or situations [82–84]. Self-initiated retrieval is thought to take place as ‘replay’ mainly occurring during hippocampal local-field potential (LFP) events called sharp-wave ripples [85–88], but also during bouts of LFP theta-band activity during running and other ongoing behaviour, resulting in ‘forward sweeps’ of hippocampal place representations ahead of the animal, at choice points in the environment [89]. Thus, during ongoing goal-directed behaviour, as well as ‘off-line’, the hippocampus has multiple modes to (re-)generate and recall information from memory, which can be flexibly used to guide decision-making and/or support consolidation [90,91].

The upshot of these recently emerging insights is to regard the hippocampal system in a broader sense than was hitherto the case, namely as a system for representing the current state of the world including the agent's, incorporating many types of causal and/or non-causal spatio-temporal relationships. The hippocampus is not passively storing episodic memories but rather actively storing information (based on synaptic plasticity) and retrieving information (by way of replay and theta-sequenced firing), where the switch between these two modes can be rapidly and flexibly made, depending on the current needs of the organism, e.g. in planning its behaviour or in consolidating previous experiences. Overall, the strong confluence of sensory thalamocortical information onto the hippocampus—as contrasted with frontal motor information which reaches it more indirectly—emphasizes that this structure is more concerned with the representation of current states of the environment and the organism, and their multisensory derivatives such as place (Where) and time (When), then being confined to the representation of an action or task space *per se*. However, the inputs to the hippocampus from the grid cells of the medial entorhinal cortex can be interpreted as representing heading direction and thus action, a source of information that in turn strongly dominates memory dynamics [72]. Having said this, the hippocampus engages as well in the representation of goal sites, and these behaviourally significant states are probably encoded with greater density and/or spatial resolution than neutral locations [68,90,92]. These goal sites play an important role in goal-directed choice but according to DAC, they need to be complemented with other information to solve the full H4W problem, and most importantly sequenced rule-based goal-oriented plans for action generated at the level of the CL.

### (c) Task representations conducive to goal-directed behaviour: prefrontal cortex

Leaving aspects of prefrontal functions in the cognitive control over memory processes in primates aside, there is accumulating evidence to cast prefrontal functions as controlling goal-directed behaviour (e.g. [93]). Whereas the hippocampus is proposed to engage in world state representations, the prefrontal cortex is more concerned with task- and action-space representations. Neurophysiological studies in rodents and primates indicate that prefrontal neurons can encode task rules that need to be followed to obtain a goal [94], individual actions or chunks of actions leading up to a goal [95–97], and goals and goal sites themselves [98,99]. Importantly, orbitofrontal and medial prefrontal-anterior cingulate neurons are sensitive to the motivational value of cues [100–104] and actions associated with goal pursuit [105,106]. Lesion studies have confirmed a causal involvement of prefrontal structures in representing goals and task rules, implying orbitofrontal cortex in reversal learning and medial prefrontal cortex in both extra-dimensional shifting [107,108] and learning action–outcome relationships [7,109]. Thus, whereas the hippocampus is proposed to represent objects and events relevant for pursuing a goal in space and time (What, When and Where), the prefrontal cortex appears better equipped to represent a task space, i.e. the set of rules, constraints, goals and goal-predictive values of cues and actions available as options to pursue goals (How) (cf. [110]). In this context, the amygdaloid complex should be included as part of a larger network for affectively driven goal-directed behaviour not only mediating value based Pavlovian response behaviours, but should also be goal-oriented instrumental behaviours which can be invigorated by Pavlovian cues [111,112].

The dominant paradigm to investigate goal-oriented decision-making emphasizes the role of the integration of perceptual evidence in terms of the firing rate given a very limited set of actions [113]. Experiments with the CL of DAC listed above (figure 1) showed that this is a rather restricted perspective because also factors such as memory, value and goals must be considered [57]. DAC theory thus suggests that numerous goals and behavioural procedures can be considered in any given task in a state-dependent fashion. This raises the question of how prefrontal cortex could keep track of this variable set of goal-oriented actions and procedures. A detailed study of the neuronal dynamics of the premotor cortex of the macaque monkey during a countermanding task has shown that the inter trial variability of the neuronal response to the movement cue is directly proportional to the errors the animal has committed (i.e. task memory) and fully predicts performance, i.e. error rate and reaction time rather than firing rate [114]. This suggests that, dependent on task memory or confidence, the neuronal dynamics allows more or less action options to compete for control and that monitoring systems regulate this process by biasing the competition between these options. This raises the question what the neural substrate is that forces a goal relevant decision among the available response options represented in this task space.

### (d) Outcome predictions and action selection mechanisms in the basal ganglia

DAC proposes that optimal decision-making depends on the integration across perceptual evidence, memory biases, values and goals [3]. Hence, the question is where in the brain such a comparison and selection could take place. The striatum (i.e. caudate-putamen and nucleus accumbens) is the main recipient of prefrontal output and is organized in different sectors, topographically laid out as a dorsolateral sector (receiving primarily sensorimotor inputs), dorsomedial sector (mainly anterior cingulate and prelimbic inputs), a ventrolateral sector (the ‘core’ of nucleus accumbens; mainly amygdaloid, prelimbic and dorsal hippocampal input) and a ventromedial sector (‘shell’; mainly prelimbic, infralimbic and ventral hippocampal input [115]). An essential organizational feature of the basal ganglia is the grouping of topographical projections in parallel ‘loops’, starting in a particular cortical area and, from there, projecting to specific striatal sectors, external segment of the globus pallidus/pallidum and output structures such as the substantia nigra reticulata [116]. By themselves, these loops do not illuminate a specific mechanism for selecting among available response options. However, striatal principal cells are connected via GABAergic recurrent collaterals, providing a potential mechanism for competitive selection [117,118]. Furthermore, the basal ganglia possesses a funnel-like structure in the sense that the downstream flow of processing in cortico-basal ganglia loops is compressed into lesser and lesser neurons. This structure may provide further competition mechanisms operating at, or in interaction with, the output levels such as substantia nigra reticulata and the internal segment of the globus pallidus [119].

By itself, the presence of GABAergic, inhibitory interactions would suggest an inflexible, learning-insensitive competition mechanism in the striatum. By contrast, recording and pharmacological studies indicate an active role of the basal ganglia in learning goal-directed behaviours. Building on previous models that framed the basal ganglia as an actor–critic architecture for (model-free) reinforcement learning [120,121], we recently argued that especially the ‘Critic’ (goal-predictive) function of the striatum is well supported by the data, whereas its implementation of the ‘Actor’ component is much less clear [9]. This deviation from a classic actor–critic scheme is based on the widespread support for reward-predictive components of cue-, action- and place-related neural coding in multiple striatal sectors (in agreement with a Critic function), whereas clear evidence for a construction in which a ‘Critic’ instructs a separate ‘Actor’ structure within the same basal ganglia is lacking [9]. When surveying the various striatal sectors, it is striking to note that striatal functions in goal prediction (usually described as ‘reward expectancy’ in animal experiments) can be attributed to all sectors, but based on domains of afferent information differing per sector. The dorsomedial striatum, for instance, has been implied in action-outcome learning [122], whereas the ventral striatal core functions in cue-outcome learning, in conjunction with its strong amygdaloid input [9,123]. By contrast, the shell of the nucleus accumbens has been implied in place-outcome learning [124]. In this scheme, the dorsolateral striatum might seem to be the ‘odd one out’ in this company, as it has been implied in habit formation and sensorimotor learning with minor or no dependence on motivational outcome. However, its role can in fact be very well accommodated if the ‘outcome’ is viewed more broadly: outcome can also be constituted by *action*, so that cue-action (or: stimulus–response) learning is subsumed under an overall basal ganglia architecture for ‘input–outcome’ learning. In conclusion, the anatomical architecture, internal wiring and information resources in afferent structures place the basal ganglia in an eminently suitable position to, first, code state–outcome relationships (where ‘state’ can be stimulus, place or action) and, second, to use this associatively learned information to force an expected outcome-dependent decision among response options represented in task space.

Outcomes (in a broad sense) of situations and actions need to be compared against predictions. The best-known candidate for expressing such as comparison—at least in the domain of reward and appetitive learning—is the reward-prediction-error mechanism that may be implemented by mesodiencephalic dopaminergic neurons projecting to striatum and prefrontal cortex [125], while the habenula has been implied in aversive learning [126]. This type of error is, however, low dimensional in nature and limited to cached value (‘I got less/more than expected’). Error signals incorporating model-based, high-dimensional information (‘I got a banana instead of the apple I expected, but they are worth the same’) may involve cortical systems such as the anterior cingulate and orbitofrontal cortex, but also striatal regions [9,127,128] (see [31] for a more detailed computational analysis of model-based versus model-free reward signals).

It is worth noting that, in a highly adaptive agent capable of planning, world and task space representations will not be static, or merely ‘slowly evolving’ as the agent moves along in search of its goals. The self-generated time-compressed replay and forward-sweeping events found in the hippocampus illustrate how past and future trajectories can be rapidly retrieved and flexibly used for planning (cf. [88,90]). These hippocampal replay events are likely to impact on the ventral striatum, where replay events have been shown to occur in succession to place-cell replay in the hippocampus [85,87]. During hippocampal forward sweeps, ventral striatal neurons code covert reward expectations, while orbitofrontal neurons code expected outcome at alternative sites that had not been selected for visiting [129,130]. Thus, especially the orbitofrontal cortex may not only provide model-based information on cue/object value before choices are made [102,103,131], but also engage in post-decisional evaluation and ‘looking back’ on previous decisions. How hippocampal events are precisely linked to prefrontal processing is less clear yet, but the medial prefrontal cortex does exhibit replay, and its activity coheres with hippocampal theta activity [132,133].

In conclusion, the framework emerging from systems and behavioural neuroscience is that goal-directed behaviour is mediated by a network of highly interconnected brain structures which directly implement H4W: (i) the hypothalamic-brain stem system functions as a key node for signalling homeostatic needs and drives grounding the ‘Why’ of goal-directed action; (ii) the hippocampus encodes episodic state representations configured in space and time, which can be rapidly retrieved online to inform and instruct decision-making systems supporting integrated representations of ‘What’, ‘When’ and ‘Where’; (iii) the prefrontal cortex encodes task space representations, comprising choice options, rules, goals, values of cues and actions relevant to obtaining the goal, and using among others information from the hippocampus and amygdala to shape these representations based on situational relevance and prior experience shaping defining potential candidates for the ‘How’ of action; and (iv) the basal ganglia impose a selection mechanism, including reward/punishment predictions as weighting factors, on the manifold options represented in frontal motor cortical structures further biasing decision-making towards the dominant goal and action defining *how* a goal can be achieved through a specific action. These systems map to components and processes of the DAC architecture: it initially deals with homeostatic needs using a reactive system but successively acquires new state and task representations as well as a new behavioural repertoire in the adaptive and CLs, which compete for selection. It is the combined contribution of elements at all these levels rather than a monolithic architectural component that realizes goal-directed behaviour.

## 3. Conclusion

In this article, we identified the central questions of goal-directed choice as ‘Why do I act? What do I need? Where and When can this be obtained, and How do I get it?’ We have analysed this H4W problem from the perspective of a system-level architecture by mapping the H4W objectives onto the DAC theory of mind and brain. Neuroscience and neuroeconomics have often benefited from a close linkage to computational methods but these often abstract from many details of situated action (e.g. model-based reinforcement learning theories [134,135] and several contributions to this special issue). By contrast, we addressed goal-directed choice from the viewpoint of a biomimetic cognitive architecture that also considers the embodied and situated aspects of the choice situation. From this perspective, the brain is a control system and its primary task is to support adaptive action in the real world rather than optimally solving abstract problems detached from perception and action. Subsequently, following the DAC framework, we proposed that the mammalian brain solves the H4W problem by integrating central representations of needs and drives (e.g hypothalamus), valence (e.g. amygdala), world and self-state spaces (e.g. neocortex and hippocampus), task space (e.g. prefrontal cortex) and multi-modal selection (e.g. basal ganglia) on the basis of multiple kinds of outcome predictions. Goal-directed choice thus results from the coherent orchestration of multiple mechanisms within a system-level architecture. That this interpretation is a reasonable approximation of the neuronal substrate of goal-oriented choice is made plausible by the fact that an existence proof of this architectural hypothesis is provided through robot-based experimentation using DAC. The DAC case studies we presented thus also illustrate how biomimetic architectures instantiated in robots can be used to explain neuronal processes and formulate predictions that bridge the gap between real-world robot behaviour and neuronal data [136].

From the DAC perspective, it can be appreciated that goal-directed selection is *multilevel*; it involves multiple mechanisms that represent information of different qualities (e.g. sensory, memory and different aspects of H4W) at different layers and engage in interactions that have to be coherently orchestrated. The DAC approach can shed light on how more complex goal-directed strategies develop on top of innate reactive control systems, too. At the lowest level, simple representations of valued states (linked to homeostatic variables) might be initially available that trigger stereotyped appetitive or aversive behaviours (e.g. following a sugar gradient in aqueous solution). These behaviours are not goal-directed because they lack key ingredients such as knowledge of causal efficacy of actions and guidance of actions using goal representation. However, they can serve to bootstrap the valuation of states of the external world (e.g. apples have value), the acquisition of goal representations at higher hierarchical levels (e.g. consumption of an apple) and associated goal-directed control strategies (e.g. reaching a specific place and climbing a ladder to obtain the apple). For example, the AL of DAC shows how during learning, Pavlovian mechanisms permit to ‘transfer’ value to novel states, e.g. visual stimuli and spatial representations that can be successively selected as goals. The CL of DAC models how state-action sequences that lead to reinforcement can be stored and increasingly support more complex goal-directed strategies [25,27]. DAC does provide *explanations* for behaviours such as observed in classical conditioning, navigation and foraging, has made testable and tested *predictions* on the underlying neuronal substrate and, in addition, has been generalized to the *control* of robotic systems. These are three key criteria of a scientific theory that should be set as the benchmarks for any theory of mind and brain. With respect to the neuronal substrate of goal-directed choice and its decomposition into the *H4W problem*, an initial mapping of the DAC taxonomy to the brain would lead to the following system-level decomposition: hypothalamus, central grey and other brain stem structures forming part of the RL self system defining Why; the amygdala as an interface between Why and What at the RL and AL level; neocortex and hippocampus forming key systems of the AL learning machinery establishing What, When and Where, with the caveat that the timing of events is probably relatively coarsely coded in hippocampus [69,70]; detailed timing of When defined through the cerebellum at the level of AL; rule-based task space construction that integrates What, Where and Why at the level of the prefrontal cortex; while the competitive processes of the basal ganglia ultimately orchestrate the How of goal-oriented action. This decomposition confirms the DAC prediction that AL systems are more closely linked to interval based real-time processing, while those of the CL are more dominated by order.

The mapping of H4W to the brain and DAC shows that goals can be seen as emerging from the foundational need systems of the physically instantiated agent. However, through the state space learning systems of the AL and the task learning systems of the CL, these goals become incrementally more abstracted from their homeostatic origins (see also [137,138]). Rather they are defined as models of states of the agent and the world that define the measurable ends of successful behavioural strategies. Hence, what we call ‘goals’ are amalgamations of sensory, affective and action states, stored in different memory systems and defined on the basis of the interaction of the agent with its varying and often conflicting needs with its dynamic environment. This incremental abstraction from needs to goals serves the transition from action in environments with continuously available sensory information that support taxon-based strategies, to tasks that depend on discontinuous and unpredictable environments with intermittent feedback. The latter case can vary from maze navigation to solving complex logical puzzles [34,139]. The predictions of this model are that any representation of sensory-affect-action states (or the DAC triad of world, self, action) that can be part of frontal cortical WM systems can become tagged as a goal state, i.e. a state at which a behavioural sequence terminates. These representations can in turn affect processing at any level of the DAC hierarchy through their ability to drive valence via specific modulatory and feedback circuits. One example would be the ability to drive the inferior olive through projections from frontal areas in this way defining the teaching signals that control plasticity at the level of the cerebellar cortex and thus its goal-based learning capabilities (e.g. [32]). This implies that goal states as defined in these terms are either explicitly tagged in order to play this role or are detected in online processing. Future work has to shed more light on this prediction.

Our analysis of goal-oriented choice, combining a neurobiological perspective with the DAC theory, exemplifies how neurorobotic and experimental methods can work hand-in-hand. As the biologically grounded model constitutes an integrated architectural solution to problems of goal-directed choice and pursuit, it can help studying the *systems-level* neurobiology of goal-directedness rather than focusing only on its components in isolation. A combined neurophysiological and neurorobotic approach holds the promise to simultaneously tackle the problem of goal-directed choice at multiple levels [140–142]: (i) the functional-behavioural level, e.g. how goals are selected and realized in the real world based on their expected value and the cost of achieving them; (ii) the mechanistic-computational level, e.g. how action outcomes are computed based on perception–action systems and how they are evaluated on-the-fly based on limited information and resources; (iii) the physiological level, e.g. how these computations are implemented in neuronal structures, focusing in particular on a system-level architecture formed by hypothalamus, hippocampus, ventral striatum, medial prefrontal cortex and amygdala; and (iv) ecology, e.g. how goal-directed abilities can be learned and adapted in real-time and in a situated environment, thus providing for a convergent multi-scale validation of theories of mind and brain. The alliance between robotics and the empirical research on the brain is beneficial for robotics, too. In developing robots endowed with goal-directed behaviour, it has considerable advantage to base them on the principles identified in biological behaviour, because central nervous systems have evolved as highly successful examples of efficient architectures enabling increasingly more sophisticated perception, cognition and behaviour. The more fundamental methodological question that the approach we sketched addresses is what shape theories of the brain will attain and how system-level questions of brain and behaviour can be effectively pursued. We argue that such answers will take the form of hybrid frameworks that integrate neurobiological research, abstract and biologically detailed computational models realized using biologically grounded real-world artefacts.

## Endnote

1 A DAC tutorial and source including replications of key studies can be downloaded from http://csnetwork.eu/education/texts-tutorials.
